# Nutrition coupled with high-load or low-load blood flow restricted exercise during human limb suspension

**DOI:** 10.1186/1550-2783-9-S1-P8

**Published:** 2012-11-19

**Authors:** Kyle J Hackney, Meghan E Everett, Lori L Ploutz-Snyder

**Affiliations:** 1 Wyle Science, Technology, and Engineering Group, Houston, Texas, USA; 2 University of Houston, Houston, Texas, USA; 3 Universities Space Research Association, Houston, Texas, USA; 4 Syracuse University, Syracuse, New York, USA

## Background

High-load resistance exercise (HRE) and low-load blood flow restricted (BFR) exercise have demonstrated efficacy for attenuating unloading related muscle atrophy and dysfunction. Protein consumption immediately before and/or after exercise has been shown to increase the skeletal muscle anabolic response to resistance training. The purpose of this study was to compare the skeletal muscle adaptations when chocolate milk intake was coupled with HRE or low-load BFR exercise during simulated lower limb weightlessness.

## Methods

Eleven subjects were counterbalanced to HRE (31 ± 14 yr, 170 ± 13 cm, 71 ± 18 kg) or low-load BFR exercise (31 ± 12 yr, 169 ± 13 cm, 66 ± 14 kg) during 30 days of unilateral lower limb suspension (ULLS); a ground based space flight analog. Both HRE and BFR completed 3 sets of supine, single leg press and calf raise exercise during ULLS. BFR exercise intensity was 20% of repetition maximum (1RM) with a cuff inflation pressure of 1.3 × systolic blood pressure (143 ± 4 mmHg). Cuff pressure was maintained during all 3 sets including rest intervals (90s). HRE intensity was 75% 1RM and was performed without cuff inflation. Immediately (<10 min) before and after exercise 8 fl oz of chocolate milk (150 kcal, 2.5g total fat, 22g CHO, 8g protein) was consumed to optimize acute exercise responses in favor of muscle anabolism. Muscle cross-sectional area (CSA), 1RM strength, and muscular endurance were determined pre and post-ULLS. Data were analyzed with condition x time (between-within) ANOVA with repeated measures using alpha of 0.05.

## Results

Unloaded limb work performed during leg press (1514 ± 334 vs. 576 ± 103) and calf raise (2886 ± 508 vs. 1233 ± 153) sessions was greater in HRE vs. BFR, respectively. Leg press training loads were 44 ± 7 kg in HRE compared to 11 ± 1 kg in BFR. Similarly, calf raise training loads were 81 ± 11 kg in HRE and 16 ± 1 kg in BFR. Pre to post-ULLS training adaptations in the unloaded leg are shown in the table below.

**Table 1:** 

	HRE (N=5)			BFR (N=6)		
	
	Pre-ULLS	Post-ULLS	%Change	Pre-ULLS	Post-ULLS	%Change
KE CSA (cm^2^)	59.2 ± 9	60.3 ± 9	+1.8	55.1 ± 4	53.7 ± 9*	-2.3
PF CSA (cm^2^)	40.1 ± 4	40.3 ± 3	+0.4	37.8 ± 2	36.0 ± 2*	-4.8
LP 1RM (kg)	57.0 ± 9	66.0 ± 12	+15.1	49.0 ± 6	43.0 ± 6*	-11.9
CR 1RM (kg)	101 ± 5	110 ± 5	+9.0	86.0 ± 7	80.0 ± 3	-6.6
LP Endurance (reps)	44.0 ± 8	39.0 ± 6	-10.0	36.0 ± 3	42.0 ± 3	+14.0
CR Endurance (reps)	30 ± 4	34 ± 5	+13.0	31 ± 2	47 ± 5*†	+51.8

*significantly different vs. pre;†significantly different vs. HRE; p < 0.05. Mean ± SE, KE= Knee Extensors, PF= Plantar Flexors, LP = Leg Press, CR = Calf Raise.

## Conclusions

When HRE is optimized for muscle anabolism during unloading muscle size and strength are preserved (or enhanced) at the expense of muscle endurance. In contrast, when BFR exercise is optimized for muscle anabolism during unloading muscle endurance is preserved (or enhanced) at the expense of muscle size and strength.

